# Degeneracy of light scattering and absorption by a single nanowire

**DOI:** 10.1038/s41598-021-98011-x

**Published:** 2021-09-20

**Authors:** Jeng Yi Lee, Yi-Huan Chen, Pai-Yen Chen

**Affiliations:** 1 Department of Opto-Electronic Engineering, National Dong Hwa University, Hualien, 974301 Taiwan; 2 Department of Electrical and Computer Engineering, University of Illinois at Chicago, Chicago, Illinois 60607 USA

**Keywords:** Optical physics, Nanophotonics and plasmonics

## Abstract

We theoretically and numerically prove that under an electromagnetic plane wave with linear polarization incident normally to a single nanowire, there exists a power diagram that could indicate scattering properties for any system configurations, material parameters, and operating wavelength. We demonstrate the distinct power distribution boundary in absorption, scattering, and extinction for a generalized nanowire with any partial wave modes dominant. In the boundary, each dominant scattering coefficients remain constant, and its energy performance would display superabsorbers or superscatterers. Interestingly, for a system with larger partial wave modes dominant, the occupied domain in the power diagram could completely cover that with lower ones. Hence, a system with different levels of partial wave modes can display the same power results, reflecting the degeneracy. This degenerate property could release more degrees of freedom in design of energy harvesting devices and sensors. We demonstrate several systems based on realistic materials to support our findings.

## Introduction

Understanding of the interaction between scatterers and impinging electromagnetic radiation is of importance for a variety of applications, such as solar energy harvesting^1–3^, strengthening interaction of light and subwavelength objects^4^, magnetic resonance imaging^5,6^, highly directivity of antennas^7–9^, sensing^10^, medical heating treatment at nanoscales^11–13^, photodetectors^14–16^, and photocatalysis^17^.

Scattering and absorption of an isotropic scatterer in extremely subwavelength dimension would have a single partial wave mode limit^18–20^. To overcome this limit, a composite subwavelength system by placing proper multi-layered configurations can induce multiple resonances at same frequency, i.e., degenerate resonances. It has demonstrated that, in the whispering gallery framework, excitation of a confined polariton can result in a superscatterer^21–23^. Analogous to degenerate resonant mechanism, minimum-scattering superabsorbers, that not only can retain minimum scattering power but also can arbitrarily strengthen absorption, was theoretically demonstrated^24,25^. With proper sizes for nanowires or metal plasmonic covers, there had experimentally boost photocurrent, photoresponse, and photocatalysis, relied on leaky mode resonances mechanism or localized surface plasmon^1,2,15–17,26^. The effort of these works is to optimize the absorption of light. On the other hand, there also demonstrated that silicon based nano-photodetection with gold coating can not only reduce charge recombination, but also reduce scattering of light contributed by dipolar and antidipolar scattering cancellation^14^, analogous to the work of cloaked sensors^10^. The central issue of the work, from energy viewpoint, is to link relation between absorption and scattering of light.

We note that due to the constraint imposed by passivity and causality, the corresponding partial absorption, scattering, and extinction cross sections are definitely bounded, irrespective of inherent system configurations, material parameters, and operating environment^27,28^. Consequently, as a benefit of the phase diagram for scattering coefficients, we can find that for each partial mode, scatterer systems can display a variety of scattering with constant absorption. However, when a scatterer system has sophisticated mode interferences, the answer to whether there can have a general power diagram valid for arbitrary scattering situations is far from obvious and remains open. The answer can definitely provide complete information in power distribution in light scattering process and address inherent limits, with potential applications in development of nano-photonics devices.

Inspired by the concept from the calculus of variations^29^, in this work, we define a energy function related to scattering and absorption, and use differential calculus to address power distribution. We theoretically and numerically prove that under an electromagnetic plane wave with linear polarization, for a cylindrical system, there exists a power diagram in which can indicate all possible power distribution, without limit of specific scattering situations. The power diagram can clearly indicate the correlation among absorption, scattering, and extinction. We also find the definite boundary of power diagram for any partial wave modes dominant. Along the boundary, the magnitudes of scattering coefficients and its phases are required the same, which corresponds to exotic superabsorbers or superscatterers. We also observe that for a system with lower partial wave modes excitation, its domain in the power diagram is just a sub-region of that with larger ones. Therefore, one can design a nanowire system, through exciting higher partial mode levels, to simulate the identical power performance occurred at lower ones by means of excitation of higher partial mode levels. We also demonstrate quasi-minimum-scattering superabsorbers and quasi-superscatterers, whose its power distribution is similar as the works^21–24^, but without degenerate resonant requirements. We believe our results can provide more degrees of freedom for nano-photonics designs in energy harvesting and sensing.

## General power diagram

Consider a cylindrically symmetric scatterer is normally impinged by a plane wave with electric field oscillated along the z-axis direction, i.e., **s** mode. The scattering, extinction, and absorption powers can be expressed in the following^18,21,30^,

1 $$\begin{aligned} P_{ext}&=P_{scat}+P_{abs}=-\frac{2}{k_0} \sqrt{\frac{\varepsilon _0}{\mu _0}}\vert E_0\vert ^2 \sum _{n=-\infty }^{\infty }\text {Re}(a_n^\mathbf{s })\nonumber \\ P_{abs}&=-\frac{2}{k_0}\sqrt{\frac{\varepsilon _0}{\mu _0}}\vert E_0\vert ^2 \sum _{n=-\infty }^{\infty }[\text {Re}(a_n^\mathbf{s }) +\vert a_n^\mathbf{s } \vert ^2] \end{aligned}$$

where $E_0$ is amplitude of incident electric field, $a_n^\mathbf{s }$ is complex-valued scattering coefficient, $k_0$ is environmental wave number, and $\varepsilon _0$ and $\mu _0$ are free space permittivity and permeability^31^. Without loss of generality, we set $\vert E_0\vert =1$ in the following analysis. We note that due to cylindrical symmetry, the scattering coefficients have a symmetry of $a_n^\mathbf{s }=a_{-n}^\mathbf{s }$. For $n=[0,1,2]$, they correspond to electric dipole, magnetic dipole, and magnetic quadrupole, respectively. The extinction power, summation of absorption and scattering power, is also related to the optical theorem, which links the scattering electric field at forward direction^32–35^. Thus, a system with non-zeros of absorption and scattering, they must possess scattering electric field along the forward direction. Under constant extinction, enhancement of absorption would reduce scattering, that would further lower the scattering intensity over all direction, but it still remains a constant scattering electric field at the forward direction^8^.

Due to the energy conservation in such a passive system, the partial absorption cross sections for each partial modes should be restricted $-[\text {Re}(a_n^\mathbf{s })+\vert a_n^\mathbf{s }\vert ^2]\ge 0$. Here $-[\text {Re}(a_n^\mathbf{s }) +\vert a_n^\mathbf{s }\vert ^2]$ is defined as normalized partial absorption power. Following this inequality, we obtain a physical bound of scattering coefficients for each partial modes, i.e., $\vert a_n^\mathbf{s }\vert \in [0,1]$ and $Arg[a_n^\mathbf{s }]\in [\frac{\pi }{2},\frac{3\pi }{2}]$^8,27^.

The normalized partial absorption power at each partial mode can not go beyond 0.25, while the upper limit for normalized partial scattering power, defined as $\vert a_n^\mathbf{s }\vert ^2$, is 1 for each partial mode. If a system has a *N* partial wave modes excited, the maximum absorption power can not be larger than $(2N+1)\times \frac{1}{2k_0}\sqrt{\frac{\varepsilon _0}{\mu _0}}$, while the corresponding scattering power would be $(2N+1)\times \frac{1}{2k_0}\sqrt{\frac{\varepsilon _0}{\mu _0}}$, corresponding to multi-channel coherent perfect absorption^36,37^. In an extreme situation, the maximum scattering power in *N* partial modes can reach $(2N+1)\times \frac{2}{k_0}\sqrt{\frac{\varepsilon _0}{\mu _0}}$, while its absorption is zero.

However, when a system is excited by multi-partial wave modes with arbitrary scattering coefficients, the answer for the power distribution in absorption, scattering, and extinction is unclear. Suppose our system has a absorption power $P_{abs}=Const.\equiv \eta$ contributed from *N* partial wave modes. Then, we want to evaluate the corresponding extreme scattering power $P_{scat}$,

2 $$P_{abs}(a_{-N}^\mathbf{s },..0,..a_{-N}^\mathbf{s })\nonumber =-\frac{2}{k_0}\sqrt{\frac{\varepsilon _0}{\mu _0}}\sum _{n=-N}^{N} {[}\text {Re}(a_n^\mathbf{s })+\vert a_n^\mathbf{s } \vert ^2]\equiv \eta.$$

The values of scattering coefficients are unknown, but there is a constraint for absorption $0\le \eta \le (2N+1)\times \frac{1}{2k_0}\sqrt{\frac{\varepsilon _0}{\mu _0}}$.

Now, we define a energy function $\textit{L}$ related to scattering and absorption powers as follows

3 $$\begin{aligned}\textit{L}(a_{-N}^\mathbf{s },..,0,..a_{N}^\mathbf{s }) &=P_{scat}(a_{-N}^\mathbf{s },..0,..a_{-N}^\mathbf{s })\nonumber \\&\qquad +\lambda P_{abs}(a_{-N}^\mathbf{s },..0,..a_{-N}^\mathbf{s })\nonumber \\&=\frac{2}{k_0}\sqrt{\frac{\varepsilon _0}{\mu _0}} \sum _{n=-N}^{N}[\vert a_n^\mathbf{s }\vert ^2-\lambda \vert a_n^\mathbf{s }\vert \cos \theta _n-\lambda \vert a_n^\mathbf{s }\vert ^2]. \end{aligned}$$

where $\lambda$ is a Lagrange multiplier and $\theta _n$ is the argument of $a_n^\mathbf{s }$.

In order to find the corresponding extreme minimum or maximum of scattering powers, the energy function $\textit{L}$ should satisfy,

4 $$\begin{aligned} \frac{\partial \textit{L}}{\partial \vert a_n^\mathbf{s }\vert } & =\frac{2}{k_0}\sqrt{\frac{\varepsilon _0}{\mu _0}}[2\vert a_n^\mathbf{s } \vert -\lambda \cos \theta _n-2\lambda \vert a_n^\mathbf{s }\vert ]=0,\nonumber \\ \frac{\partial \textit{L}}{\partial \theta _n} & =\frac{2}{k_0}\sqrt{\frac{\varepsilon _0}{\mu _0}} {[}\lambda \vert a_n^\mathbf{s }\vert \sin \theta _n]=0. \end{aligned}$$

that are valid for $n=-N$ to *N*. In the latter expression, to obtain non-trivial solutions for $\lambda$ and $a_n^\mathbf{s }$, the solutions should be $\theta _n=0$ or $\theta =\pi$. However, due to non-gain materials embedded, the applicable solution would be $\theta _n=\pi$. By using this outcome and the first expression in Eq. (4), we have $\vert a_n^\mathbf{s }\vert =\frac{\lambda }{2\lambda -2}\equiv s$. Further, $a_n^\mathbf{s }=-s$ represents that at the extreme scattering by constant absorption, the magnitudes of scattering coefficients are identical and the corresponding phases are $\pi$, for each partial wave modes. Consequently, we can express the corresponding scattering coefficients from Eq. (2),

5 $$\begin{aligned} a_n^\mathbf{s }=\frac{-1\pm \sqrt{1-\frac{2\eta k_0}{(2N+1)} \sqrt{\frac{\mu _0}{\varepsilon _0}}}}{2}. \end{aligned}$$

In the square root, it can guarantee a non-negative value, because $0 \le \eta \le \frac{2N+1}{2k_0}\sqrt{\frac{\varepsilon _0}{\mu _0}}$. The ultimate absorption corresponds to multi-channel coherent perfect absorption. More appealingly, our results imply that under a constant absorption, there can have two extreme scattering powers:

6 $$\begin{aligned} \mathbf{Max} [{P_{scat}}]&=\frac{1}{k_0}\sqrt{\frac{\varepsilon _0}{\mu _0}} (2N+1)\left[ 1+\sqrt{1-\frac{2\eta k_0}{(2N+1)} \sqrt{\frac{\mu _0}{\varepsilon _0}}}\right. \nonumber \\&\quad \left. -\frac{\eta k_0}{(2N+1)} \sqrt{\frac{\mu _0}{\varepsilon _0}}\right] ,\nonumber \\ \mathbf{Min} [{P_{scat}}]&=\frac{1}{k_0} \sqrt{\frac{\varepsilon _0}{\mu _0}}(2N+1) \left[ 1-\sqrt{1-\frac{2\eta k_0}{(2N+1)} \sqrt{\frac{\mu _0}{\varepsilon _0}}}\right. \nonumber \\&\quad \left. -\frac{\eta k_0}{(2N+1)} \sqrt{\frac{\mu _0}{\varepsilon _0}}\right] . \end{aligned}$$

**Figure 1:**
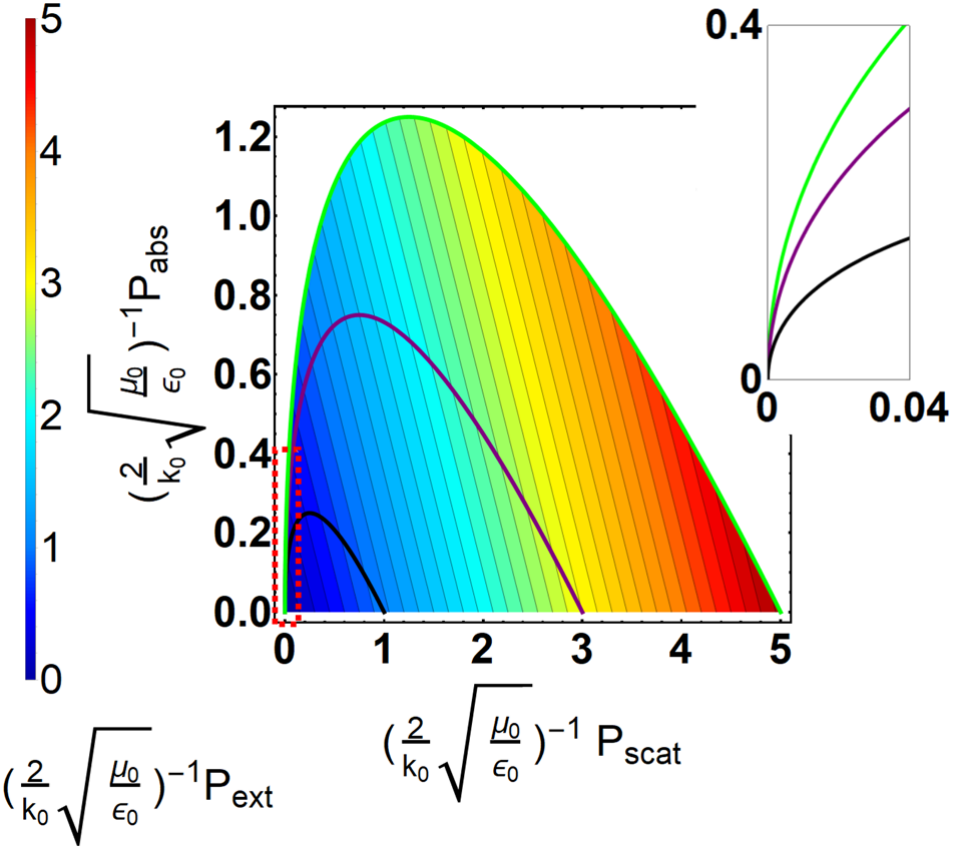
Power contour diagram for normalized absorption, scattering, and extinction powers. The black line indicates the boundary for a system with $N=0$ mode dominant, the purple is for $n=[-1,0,1]$ modes, and the green is for $n=[-2,-1,0,1,2]$ modes. The red dashed box highlights a region with a lower scattering, but with overwhelming absorption. Inset shows the detailed power distribution among absorption and scattering for the corresponding red dashed box.

Based on Eq. (6), by tuning $\eta$, we can depict a clear boundary for scattering and absorption as N partial modes are excited, as shown in Fig. 1. Inside the boundary, by $P_{ext}=P_{scat}+P_{abs}$, we can make a contour plot to indicate extinction. Here $N=0$ represents a system with only electric dipole mode supported, $N=1$ has electric and magnetic dipoles modes, and $N=2$ has electric dipole, magnetic dipole, and magnetic quadrupole modes. We can see that for a system supported by N modes, the occurrence of maximum normalized extinction accompanies with maximum of normalized scattering, while the corresponding normalized absorption is zero, corresponding to superscattering^21–23^. Additionally, along with a constant extinction, increasing absorption would reduce scattering. We will discuss this anomalous scatterer in the following analysis.

We note that for a system with N partial modes dominant, the corresponding region in the power diagram is just a sub-region of a system with higher modes. As a result, the scattering system can display the same energy performance, but its inherent scattering coefficients can be completely different.

## Quasi-minimum-scattering- superabsorbers and quasi-superscatters

**Figure 2:**
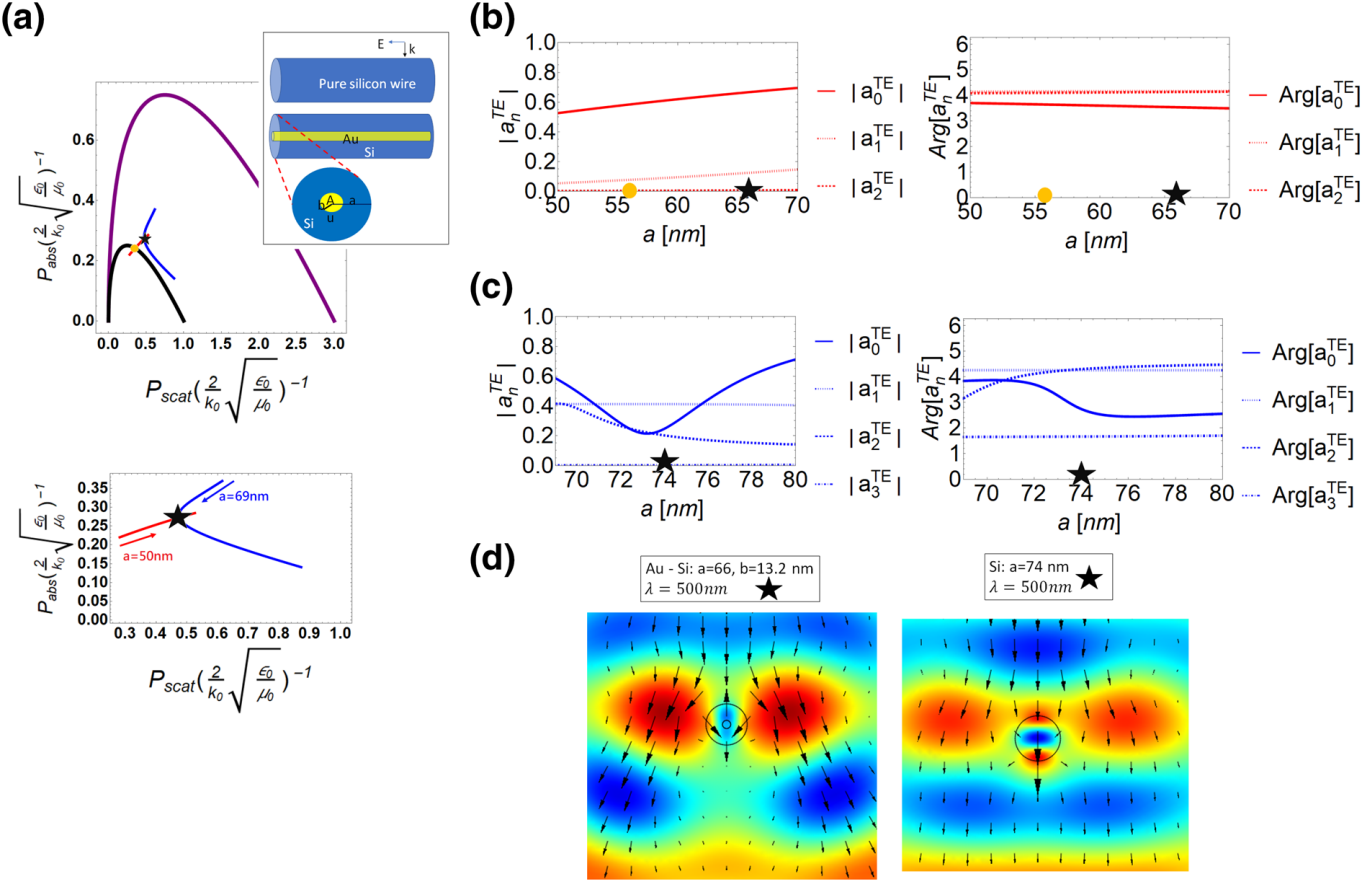
Power distributions of two different systems by tuning sizes are shown in the power diagram (**a**). Insert shows these two systematic configurations. Red line is for a gold-silicon core-shell system. Here the radius ratio (inner to outer) is fixed constant, 0.2. Blue line is for a homogeneous silicon system. When tuning $a=50$ nm to 70 nm of a gold-silicon core-shell system, the corresponding power distribution shown in the red line, goes through $N=0$ to $N=1$ region. An orange dot denotes that the system is cross boundary of $N=0$. In the blue line, it shows that the size of silicon nanowire is tuned from 69 to 80 nm. An intersection of red and blue lines (marked by a black star) denotes two systems have same power in absorption, scattering, and extinction. In below (**a**), it reveals the detail of power distribution for the red and blue lines. In (**b**) and (**c**), we study the constitution of dominant scattering coefficients for the gold-silicon core-shell and homogeneous silicon systems in magnitudes and phases, respectively. Here the relative permittivity of gold we use is $-2.81 + i3.19$ and that of silicon is $18.5+ i0.63$. The operating wavelength is 500 nm. In (**d**), we plot the electric field distribution for two different systems with the same power distribution in scattering, absorption, and extinction, corresponding to black stars in (**b**) and (**c**).

To verify our findings, in Fig. 2, we first discuss systems with realistic materials embedded^38^. We design two types of systems operated at the wavelength of 500 nm, but with different geometrical sizes, materials, and inherent configurations. In Fig. 2a, the red line represents that a core-shell nanowire system is constituted by gold in shell and silicon in core, while the blue line is a homogeneous silicon nanowire (see the insert of (a) for schematics of both scattering events). We tune the outer radius *a* from 50 to 70 nm for the core-shell system, while the ratio of shell-core radii is fixed constant 0.2. For the homogeneous silicon nanowire, we tune the radius from 69 to 80 nm. Initially, the red line is inside $N=0$ region when the outer radius is 50 nm. When the outer radius is larger than 55 nm (marked by a yellow dot in Fig. 2a), its power distribution then goes from $N=0$ through $N=1$ region. In order to understand its underlying mechanism, we study the magnitudes and phases of the dominant scattering coefficients in Fig. 2b. We can observe that the dominant mode in gold-silicon core-shell system is $N=0$ (electric dipole) when $a=50$ nm, however, with increasing system size *a*, the $N=1$ (magnetic dipole) mode would gradually become another primary contribution. We also find that the phase of the electric dipole mode is nearly $\pi$ at $a=55$ nm when close to the boundary of $N=0$, as expected in Fig. 2b.

In the silicon nanowire system, we depict a blue line in Fig. 2a by tuning geometry size from 69 to 80 nm. We also perform the mode analysis with $n=[0,1,2,3]$ in Fig. 2c, which the system obviously belongs to $N=2$ region. This outcome reveals that the domain of this silicon system should be $N=2$, but its location is at $N=1$, which reflects the degeneracy of light scattering. Moreover, when the radius of silicon system is 74 nm marked by a black star, we find the absorption, scattering, and extinction, are identical to that of the gold-silicon core-shell system with the radius 66 nm, which can be readily understood by comparing the electric field distributions shown in Fig. 2d. Although their constitutions of scattering coefficients are totally different, they can provide the same power performances in light scattering.

**Figure 3:**
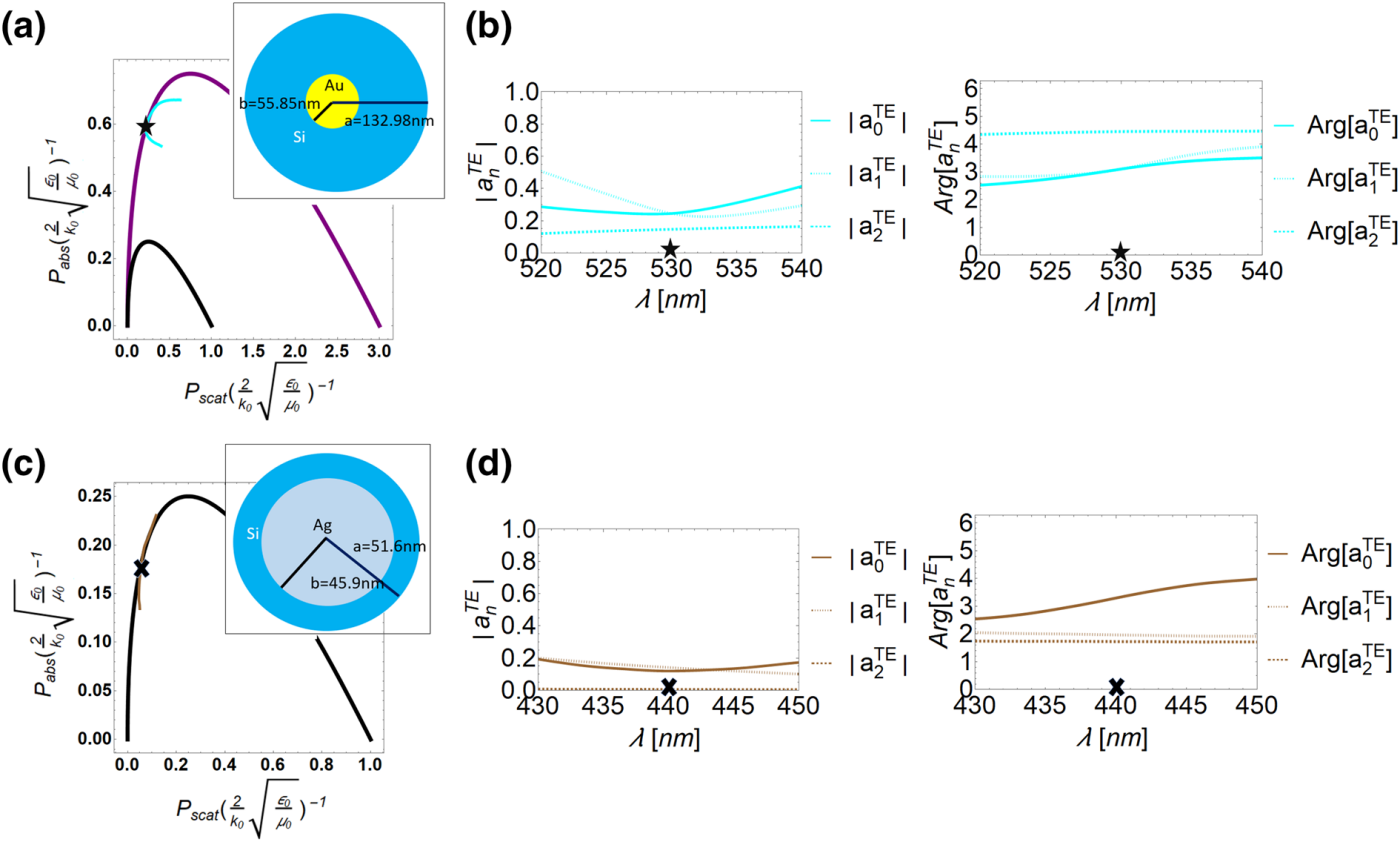
Power distributions of two different systems by tuning operating wavelength in (**a**) and (**c**), respectively. In (**a**), the cyan line represents the gold-silicon core-shell system with an outer radius of 132.98 nm and a ratio of the inner to outer radius of 0.42. The corresponding magnitudes and phases of the scattering coefficients in this gold-silicon system are shown in (**b**). The operation wavelength in this system is from 520 to 540 nm. An intersection of the $N=1$ boundary is occurred at operating wavelength 530 nm, marked by a black star. In (**c**), this system is made of silver-silicon core-shell with outer radius of 51.6 nm and ratio of outer-inner radius of 0.89. The operating wavelength for this system is from 430 to 450 nm. An intersection of the $N=0$ boundary is at 440 nm, marked by a black cross. The magnitudes and phases of dominant scattering coefficients in this operating wavelength window are shown in (**d**).

Next, we discuss the existence of the minimum-scattering superabsorbers^24,25^, in which the system can absorb more energy while maintaining scattering by exciting more partial modes, as highlights in a red-dashed box of Fig. 1. We consider a core-shell nanowire system made of silicon in shell and gold in core (see the inset of Fig. 3a), where the ratio of inner to outer radius is fixed 0.42 and the outer radius is 132.98 nm. The material dispersions are based on measurement results provided in^38^. In Fig. 3a, we study power distribution of the core-shell system with varying operation wavelength from 520 to 540 nm, as denoted by cyan color line. To understand its inherent scattering coefficient components, we also plot the magnitudes and phases for each partial modes $n=[0,1,2]$, as shown in Fig. 3b. In this wavelength range, the dominant modes are $n=[0,1]$. We note that only at 530 nm, the cyan line would intersect with the boundary $N=1$ marked by a black star, revealing a system with lower scattering but larger absorption. In this case, the normalized absorption and normalized scattering power are [0.6, 0.2], respectively. In phase analysis of Fig. 3b, it is interesting to see that the phases from dominant modes at 530 nm would be $\pi$, marked by a black star.

Furthermore, we consider another system of silicon in shell and silver in core with an outer radius of 51.6 nm and the ratio of inner to outer radii 0.89 (see the inset of Fig. 3c). Figure 3c shows a brown color line in the wavelength range from 430 to 450 nm. Here the system obviously is operated in the $N=0$ region. We mark the system operated at 440 nm by a black cross, an intersection at $N=0$ boundary, with the minimum-scattering superabsorption property. The normalized absorption and normalized scattering power in this case are [0.18, 0.05], respectively. However, when we study the components of dominant scattering coefficients in Fig. 3d, we find the contributed modes are $n=0$ and $n=1$. In the phase analysis of Fig. 3d, the phases of dominant modes are not needed to be $\pi$. We note that this system certainly possesses the desirable power performance for a minimum-scattering superabsorber. This outcome reveals that when designing a system related to energy issue, it can have an opportunity to relax constraints of scattering coefficients when taking a system with higher partial modes into account.

**Figure 4:**
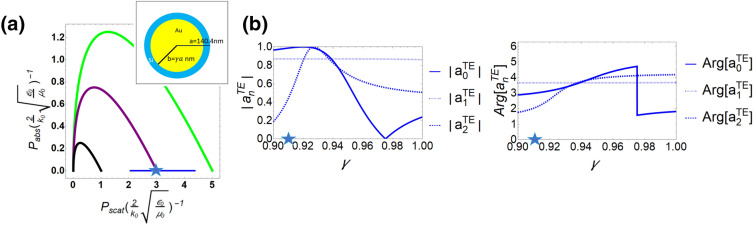
(**a**) Power distribution of a gold-silicon shell-core system by tuning ratio of inner to outer radius, $\gamma$, is shown in blue line. Here we ignore the material losses, but the real part of permittivities are based on data^38^. With outer radius of 140.4 nm and operating wavelength of 500 nm, we tune ratio of inner to outer radius $\gamma$ from 0.9 to 1. In a blue star, the system can display a superscattering property for $N=1$. The magnitudes and phases of dominant scattering coefficients for this system are shown in (**b**). Here the relative permittivities for lossless gold is $-2.8$ and for lossless silicon is 18.5.

In a superscattering case, the system has multiple partial modes resonances operated at same wavelength, beyond a single mode limit. This mechanism results from inducing confined surface waves in multi-layered system^21–23^. Here we relax this constraint by employing more higher modes excited. In Fig. 4, we choose a core-shell system with gold in shell and silicon in core by tuning a ratio of inner to outer radius, $\gamma$ (see the inset of Fig. 4a). The operating wavelength is fixed to 500 nm. We assume that the materials in this system are lossless, in order to clearly observe its mode resonances. The blue line denotes the system with $\gamma =0.9$ to $\gamma =1$. We can observe that when $\gamma =0.912$ (marked by a blue star), there has an intersection with $N=1$ boundary, but its dominant modes are $n=[0,1,2]$ as shown in Fig. 4b. Moreover, its phases are not $\pi$. This case displays a superscattering result, but it is achieved by exciting higher partial wave modes, i.e., quasi-superscatters. We note that with state of the art, hetero-nanowires can have Co–Ag/Pt core–shell^39–41^, Fe-Au/Ag core–shell^39,42,43^, In-Si core-shell^39,44^, Au–Co core–shell^39,45^ and GaP-ITO/Ag/Cu core-shell^46^. We believe our findings in quasi-minimum-scattering- superabsorbers and quasi-superscatters might be demonstrated with these already structures.

## Conclusion

With an employed energy function related to scattering and absorption, and the use of differential calculus and Lagrange multiplier, we propose the general power digram involving scattering, absorption, and extinction, for any passive cylindrical systems. The power diagram is irrespective of any structure configuration, any materials, and any operating environment. For a system with N partial wave modes supported, there has a clear boundary in absorption, scattering, and extinction. Along the boundary, all dominant scattering coefficients are the same. However, inside the boundary, there has no such relation for scattering coefficients. The region of a system with higher partial modes can completely cover a system with lower modes. Therefore it can properly induce higher modes to simulate the same power distribution in lower ones. This is a degeneracy of light scattering in absorption, scattering, and extinction. We discuss several examples of quasi-minimum -scattering superabsorption and quasi-superscattering. This work not only provides the complete information for power distribution in light scattering, but also relaxes degrees of freedom in practical design of power harvesting and sensing.

## References

[1] Cao L (2010). Semiconductor nanowire optical antenna solar absorbers. Nano Lett..

[2] Garnett EC, Brongersma ML, Cui Y, McGehee MD (2011). Nanowire solar cells. Ann. Rev. Mater. Res..

[3] Brongersma ML, Cui Y, Fan S (2014). Light management for photovoltaics using high-index nanostructures. Nat. Mater..

[4] Kneipp K (1997). Single molecule detection using surface-enhanced Raman scattering (SERS). Phys. Rev. Lett..

[5] Slobozhanyuk AP (2016). Enhancement of magnetic resonance imaging with metasurfaces. Adv. Mater..

[6] Rizza C, Palange E, Alecci M, Galante A (2020). Mimicking localized surface plasmons via mie resonances to enhance magnetic-resonance-imaging applications. Phys. Rev. Appl..

[7] Arslanagić S, Ziolkowski RW (2018). Highly subwavelength, superdirective cylindrical nanoantenna. Phys. Rev. Lett..

[8] Lee JY, Miroshnichenko AE, Lee R-K (2017). Reexamination of Kerkers conditions by means of the phase diagram. Phys. Rev. A.

[9] Lee JY, Miroshnichenko AE, Lee R-K (2018). Simultaneously nearly zero forward and nearly zero backward scattering objects. Optics Express.

[10] Alù A, Engheta N (2009). Cloaking a sensor. Phys. Rev. Lett..

[11] Huang X, Jain PK, El-Sayed IH, El-Sayed MA (2008). Plasmonic photothermal therapy (PPTT) using gold nanoparticles. Lasers Med. Sci..

[12] Tribelsky MI, Miroshnichenko AE, Kivshar YS, Lukyanchuk BS, Khokhlov AR (2011). Laser pulse heating of spherical metal particles. Phys. Rev. X.

[13] Hirsch LR (2003). Nanoshell-mediated near-infrared thermal therapy of tumors under magnetic resonance guidance. Proc. Natl. Acad. Sci..

[14] Fan P (2012). An invisible metal-semiconductor photodetector. Nat. Photonics.

[15] Cao L, Park J-S, Fan P, Clemens B, Brongersma ML (2010). Resonant germanium nanoantenna photodetectors. Nano Lett..

[16] Takiguchi M (2020). Hybrid nanowire photodetector integrated in a silicon photonic crystal. ACS Photonics.

[17] Ramadurgam S, Lin T-G, Yang C (2014). Aluminum plasmonics for enhanced visible light absorption and high efficiency water splitting in core-multishell nanowire photoelectrodes with ultrathin hematite shells. Nano Lett..

[18] Bohren CF, Huffman DR (2008). Absorption and Scattering of Light by Small Particles.

[19] Hamam RE, Karalis A, Joannopoulos J, Soljačić M (2007). Coupled-mode theory for general free-space resonant scattering of waves. Phys. Rev. A.

[20] Tribelsky MI, Lukyanchuk BS (2006). Anomalous light scattering by small particles. Phys. Rev. Lett..

[21] Ruan Z, Fan S (2010). Superscattering of light from subwavelength nanostructures. Phys. Rev. Lett..

[22] Qian C (2019). Experimental observation of superscattering. Phys. Rev. Lett..

[23] Ruan Z, Fan S (2011). Design of subwavelength superscattering nanospheres. Appl. Phys. Lett..

[24] Estakhri NM, Alu A (2014). Minimum-scattering superabsorbers. Phys. Rev. B.

[25] Mann SA, Garnett EC (2013). Extreme light absorption in thin semiconductor films wrapped around metal nanowires. Nano Lett..

[26] Qin X (2021). Surface plasmon-photon coupling in lanthanide-doped nanoparticles. J. Phys. Chem. Lett..

[27] Lee JY, Lee R-K (2016). Phase diagram for passive electromagnetic scatterers. Optics Express.

[28] Miroshnichenko AE, Tribelsky MI (2018). Ultimate absorption in light scattering by a finite obstacle. Phys. Rev. Lett..

[29] Landau LD, Lifschic E (1978). Course of theoretical physics. vol 1: Mechanics.

[30] Lee JY, Chung Y-H, Miroshnichenko AE, Lee R-K (2019). Linear control of light scattering with multiple coherent waves excitation. Optics Lett..

[31] By electromagnetic duality, the related formulas for tm, i.e., magnetic field is along z axis, could be obtained by $$\varepsilon \rightarrow \mu$$, $$\mu \rightarrow \varepsilon$$, $$\vec{E}\rightarrow \vec{H}$$, and $$\vec{H}\rightarrow -\vec{E}$$. Advanced engineering electromagnetics (CA Balanis, John Wiley & Sons, 1999).

[32] Newton RG (1976). Optical theorem and beyond. Am. J. Phys..

[33] Jackson, J. D. Classical electrodynamics (1999).

[34] Alù A, Engheta N (2010). How does zero forward-scattering in magnetodielectric nanoparticles comply with the optical theorem?. J. Nanophoton..

[35] Liu W, Kivshar YS (2018). Generalized Kerker effects in nanophotonics and meta-optics. Optics Express.

[36] Noh H, Chong Y, Stone AD, Cao H (2012). Perfect coupling of light to surface plasmons by coherent absorption. Phys. Rev. Lett..

[37] Bai P, Wu Y, Lai Y (2016). Multi-channel coherent perfect absorbers. EPL Europhys. Lett..

[38] Palik ED (1998). Handbook of Optical Constants of Solids.

[39] Kim S, Kim J-M, Park J-E, Nam J-M (2018). Nonnoble-metal-based plasmonic nanomaterials: Recent advances and future perspectives. Adv. Mater..

[40] Wang L (2011). Plasmonics and enhanced magneto-optics in core-shell Co-Ag nanoparticles. Nano Lett..

[41] Wang L (2017). Plating precious metals on nonprecious metal nanoparticles for sustainable electrocatalysts. Nano Lett..

[42] Kayal S, Ramanujan RV (2010). Anti-cancer drug loaded iron-gold core-shell nanoparticles (Fe@ Au) for magnetic drug targeting. J. Nanosci. Nanotechnol..

[43] Wang L (2010). Localized surface plasmon resonance enhanced magneto-optical activity in core-shell Fe-Ag nanoparticles. J. Appl. Phys..

[44] Cingarapu S, Yang Z, Sorensen CM, Klabunde KJ (2011). Synthesis of indium nanoparticles: Digestive ripening under mild conditions. Inorganic Chem..

[45] Toal B (2014). Optical and magneto-optical properties of gold core cobalt shell magnetoplasmonic nanowire arrays. Nanoscale.

[46] Lin T, Ramadurgam S, Yang C (2017). Design of contact electrodes for semiconductor nanowire solar energy harvesting devices. Nano Lett..

